# Is leishmaniasis adequately notified in Sri Lanka? A survey among doctors from an endemic district, Sri Lanka

**DOI:** 10.1186/s12889-020-09066-w

**Published:** 2020-06-12

**Authors:** Chandana Hewawasam, Hema S. Weerakoon, Vyshnavi Thilakan, Tishni Lelwala, Kalana Prasanka, A. S. Rathnayaka, Shanika Gamage, Suneth Agampodi

**Affiliations:** 1 Department of Physiology, Faculty of Medicine and Allied Health Sciences, Rajarata University, Saliyapura, Sri Lanka; 2 Medical Officer/Public Health, Office of the Provincial Director of Health Services, Anuradhapura, Sri Lanka; 3 Department of Community Medicine, Faculty of Medicine and Allied Health Sciences, Rajarata University, Saliyapura, Sri Lanka; 4 Office of the Regional Director of Health Services, Anuradhapura, Sri Lanka

**Keywords:** Leishmaniasis, Notification, Physicians, Knowledge, Attitude, Practice, Sri Lanka

## Abstract

**Background:**

Leishmaniasis is a notifiable disease in Sri Lanka since 2008. Previous studies show a gap in the notification of leishmaniasis. The purpose of the present study was to determine the Knowledge, attitudes and practice of medical officers regarding leishmaniasis.

**Methods:**

A cross-sectional study was conducted in the Anuradhapura district which reported the highest case load of leishmaniasis. Medical officers from public and private health care institutes in the area filled a self-administered questionnaire in the presence of the investigators.

**Results:**

One hundred and eighty-eight (188) medical officers completed the questionnaire. Of them, 95.7% were aware of leishmaniasis as a parasitic infection and 84.7% correctly identified *Leishmania donovani* as the causative organism in Sri Lanka. From the respondents, 181 (96.8%) knew that the vector of leishmaniasis is sand fly. Cutaneous leishmaniasis was reported as the most prevalent form of leishmaniasis in the country by 176 (94.1%). Nearly half of the respondents (98, 54.1%) were aware of the fact that the Anuradhapura district has the highest disease burden. Many of them had the idea that leishmaniasis is an emerging disease (155, 84.3%,) and early diagnosis is important in controlling the disease (163, 89.1%). Although about three fourth (123, 73.7%,) of the participants mentioned that leishmaniasis should be notified at first clinical suspicion, only 74 (42.5%) were aware that it is a legal requirement. Some medical officers (39, 22%) believed that the current notification system in the country is not effective. Unavailability of notification forms (60, 36.8%) heavy workload (85, 50.3%) and inadequate supportive staff (55, 35.1%) were reported as barriers for timely notification. Even though 105 (58.0%) of medical officers had suspected leishmaniasis during the last 8 years period only 35 (19.4%) had notified.

**Conclusions:**

Even though more than 90% of the participants had good theoretical knowledge about leishmaniasis; notification of leishmaniasis is considerably inadequate. This study emphasizes the need for greater efforts to improve the notification of leishmaniasis in Sri Lanka.

## Background

Leishmaniasis is an emerging but neglected parasitic disease caused by species of genus *Leishmania* which is transmitted during the bite of an infected phlebotomine sand fly. It is known to manifest in 3 main forms; cutaneous leishmaniasis, mucocutaneous leishmaniasis and visceral leishmaniasis in humans [1]. About 20 species or subspecies have been identified in relation to leishmaniasis in humans. Cutaneous leishmaniasis is usually caused by *L.tropica* and *L.major; L.braziliensis* and *L.panamensis* are responsible for mucocutaneous leishmaniasis. Visceral leishmaniasis is usually a result of *L.donovani* and *L.infantum* infections [2]. Inside the reticuloendothelial system of infected individuals, organisms multiply and liberate amastigotes into the blood. These amastigotes enter into the sand fly’s gut during a bite and multiply into promastigotes which can be transmitted into a new host [3].

Cutaneous leishmaniasis is characterized by single or multiple crusted painless papules usually found in exposed areas of the body. Extensive mid facial destruction, tissue overgrowth obstructing the nares, septal granulation and gingivitis are features of mucocutaneous leishmaniasis. Kala-azar or Visceral leishmaniasis causes a systemic illness with fever, weight loss, hepatosplenomegaly and pancytopenia [4]. While visceral leishmaniasis is the most fatal form of the disease, cutaneous leishmaniasis has been identified as the most prevalent form [5].

Diagnosis of leishmaniasis is mainly clinical; this can be confirmed by isolating the parasite in the skin smears or biopsies taken from the lesions. Detection of antibodies to recombinant rK 39 antigen in patients serum and polymerase chain reaction (PCR) assays can also be used in diagnostic purposes [1, 6]. Intralesional, intramuscular or intravenous sodium stibogluconate (SSG) injections and cryotherapy with liquid nitrogen are the main treatment modalities for leishmaniasis [6]. Nevertheless, cutaneous leishmaniasis may heal even without treatment. Applying repellents on the exposed body areas, usage of insecticide-treated bed nets and wearing long-sleeved dresses when staying outdoor are some of the measures to prevent the bite of a sand fly. Identifying infected patients and early treatment is of greater importance in ceasing the spread of the disease.

Epidemiology of the leishmaniasis depends on several factors related to the life cycle of the parasite, human behavior, and climate. The tropical climate facilitates the breeding of sand flies. Traveling to endemic countries and urbanization invading forest lands increase the chance of humans getting bitten by vectors. Poor socio-economical status increases the risk of leishmaniasis. Malnutrition compromises the immunity aggravating the self-limiting disease into full-blown systemic illness. Poor housing and sanitary conditions (such as a lack of waste management or open sewerage) may increase sand fly breeding and resting sites, as well as their access to humans. Zoonotic transmission can occur in livestock farming, by rodents or by domestic pets such as dogs. Crowded houses attract sand flies providing good sources for their blood-meals. Human behaviors, such as sleeping outside or on the ground, may increase the risk of sand fly bites [3, 7, 8].

Around 0.7 to 1 million new leishmaniasis cases are diagnosed every year in the world [7]. Disease affected regions include Africa, America, the East Mediterranean region, Europe and South-East Asia [5]. Annual death count due to leishmaniasis is 26,000 to 65,000 across the globe [7]. Cutaneous leishmaniasis is the most prevalent form of the disease found in Sri Lanka and the causative organism has been identified as *L*.*donovani,* the organism which is mainly responsible for visceral leishmaniasis in the globe [9]. The North Central province of the country is a semi-urban area with a tropical climate. According to the latest annual health bulletin published by the ministry of health Sri Lanka, Anuradhapura district recorded the second-highest number of cutaneous leishmaniasis cases (277 cases, 22%) in the country [10]. (But at the time of data collection the highest case load was from Anuradhapura district for six consecutive years) [11].

There are evidence for gap between actual figures and the number of cases reported. According to a survey done in Mulathiv district of the northern province between 2011 and 2013, more than 200 cases of Leishmaniasis among military recruits had been missed from the national disease surveillance reports [12]. This suggests that there is a problem in the notification system of the country. Knowledge, attitude, and practices (KAP) of medical officers play an important role in infectious disease control in any setting. Knowledge about the disease is vital in making the right diagnosis, carrying out the relevant investigations and treating the patients. Good attitudes together with good practices such as timely notification to the relevant authorities would ensure effective control of the disease before growing into a pandemic.

However, there is no published data on studies done in the country regarding knowledge, attitude, and practices of medical officers related to leishmaniasis. Knowing the KAP of medical officers related to leishmaniasis helps to identify the obstacles in controlling the disease and carrying out remedial measures. The main objective of this study was to assess the Knowledge, attitude, and practices of medical officers about leishmaniasis and notification in a MOH area (a health unit headed by **M**edical **O**fficers of **H**ealth) in Anuradhapura district.

## Methods

### Study area and design

A cross-sectional study was carried out in Nuwaragampalatha East MOH. This MOH area was selected as it caters to the highest number of doctors per MOH area in the Anuradhapura district. The estimated population of this MOH area is 75,326. Anuradhapura district is in the dry zone of the country which has a tropical climate with usual temperature ranging from 20 to 35 °C. Except in the town area the rest of the land has good forest cover parts of which have now been converted to cultivation fields called “chena”. Paddy cultivation and “chena” cultivation are the main modes of income of the people living in the rural areas of Anuradhapura district. These cultivations mainly depend on monsoon rain which falls between December to February.

### Sample size and sampling techniques

The sample size was calculated using the *n* = Z^2^P (1-P)/d^2^ (a world health organization (WHO) recommended statistical formula for health studies) where *n* = number of study subjects (medical officers) enrolled in the study, *Z* = test statistic which allows calculating result with 95% confidence (1.96), *P* = expected proportion in the population-based on previous studies, (as this was unknown we kept *p* = 0.5 and d = absolute error or precision as 8% (for 50% prevalence). *n* = 150. All the medical officers (334) in the selected MOH area were included in the study. Lists of medical officers were obtained from the registers maintained at the respective hospital and Provincial director of health services office. To be included in the study, participants must have had a Bachelor of Medicine, Bachelor of Surgery (M.B.B.S.) or Doctor of Medicine (MD) or equivalent degree and be employed in a government or a private hospital. Other health care workers were excluded from the study as they are not directly involved in diagnosing and notifying the disease.

### Data collection

The data was collected during May 2016 to February 2017 using a self-administered, structured questionnaire developed by the investigators based on the “Leishmaniasis fact sheet” published by the WHO and “surveillance case definitions for notifiable diseases in Sri Lanka” booklet published by epidemiology unit, Sri Lanka (Additional file 1) [7, 13]. Investigators visited the medical officers at their working units and got the questionnaires filled by them in the presence of investigators. All participants provided informed written consent before filling the questionnaire. It took about 15 min to fill the questionnaire by the respondents.

The questionnaire was comprised of four parts. Part A related to study subjects socio-demographic details, Part B on knowledge regarding leishmaniasis and notification, Part C on attitude towards leishmaniasis and notification and Part D on practice related to leishmaniasis and notification. Knowledge was assessed using a 15-item questionnaire. Disease prevalence, causative organisms, mode of transmission and vector, clinical features and treatment methods were assessed under this section. Attitudes were assessed using a 7-item questionnaire and the section on practice had 8 items. The study protocol was reviewed and approved by the ethics review committee of the faculty of Medicine and Allied Sciences, Rajarata University, Sri Lanka.

### Data management and analysis

Data were double entered and analyzed using statistical package for social sciences (SPSS) version 21 software. Descriptive statistics were used to explain the characteristics of the sample such as age, sex and service experience. Multiple linear regression model was applied to analyze the effects of predictor variables on the scores. The frequency distributions were calculated to analyze the rest of the components.

## Results

One hundred and eighty-eight (188) medical officers from eight health care institutions completed the questionnaire making a response rate of 53.9%. The majority of the respondents were from the Teaching hospital Anuradhapura (152, 82.2%) which is the largest hospital in the selected MOH division. Demographic details and characteristics of the study population are summarized in Table 1.

**Table 1:** Characteristics of the respondents

Characteristics	No. (%) of Doctors (*N* = 188)	Mean (SD)
Male	115 (61.5)	
Age		35.65 (8.95)
≤ 35	115 (61.5)	
36–45	41 (21.9)	
46–55	23 (12.3)	
≥ 56	8 (4.3)	
Service years		7.47 (7.89)
≤ 5	105 (57.4)	
6–15	50 (27.3)	
16–25	20 (10.9)	
≥ 26	8 (4.4)	
Current position		
Grade MO	119 (64)	
Intern MO	34 (18.3)	
Registered MO	17 (9.1)	
Consultant	10 (5.4)	
Private practitioner	4 (2.2)	
Registrars	2 (1.1)	

(Total may not always sum to N due to missing data)

*SD* Standard deviation, *MO* Medical officer

More than 95 % of the participants (177, 95.7%) were aware that leishmaniasis is a parasitic infection and 84.7% (155) correctly identified *L.donovani* as the causative organism in Sri Lanka. From the respondents, 96.8% (181) knew that the vector of leishmania is sand fly. Cutaneous leishmaniasis was reported as the most prevalent form of leishmaniasis in the country by 94.1% (176). Nearly half of the study population (98, 54.1%,) was aware of the fact that the Anuradhapura district has the highest disease burden.

Skin ulcer was known by 86.6% (162) as the commonest symptom of cutaneous leishmaniasis and 52.7% (97) knew that skin lesions are painless and non-itching. One hundred and twenty-five respondents (67.2%) agreed with the statement “diagnosis of cutaneous leishmaniasis is mainly clinical”. Majority of the participants (101, 68.7%) selected intravenous/intramuscular Antimonals as the treatment option for leishmaniasis but only 31(17%) doctors knew cutaneous leishmaniasis may be cured even without treatment.

Although about three fourth (123, 73.7%,) of the participants mentioned that leishmaniasis should be notified at first clinical suspicion, only 42.5% (74) were aware that it is a legal requirement under the court of law in Sri Lanka. One hundred and fifty-two participants (84%) reported that all medical officers can notify the disease. Only forty-three (28.9%) medical officers knew the correct notification chain. Table 2 shows respondents’ attitudes regarding leishmaniasis and the notification system. Participants were questioned about their involvement in notifying a suspected case of leishmaniasis and data are summarized in Table 3. Even though 58.0% (105) of medical officers had suspected leishmaniasis during the last 8 years period only 19.4% (35) had notified. Heavy workload (85, 50.3%), unavailability of notification forms (60, 36.8%), and inadequate supportive staff (55, 35.1%) were reported as barriers for timely notification. According to the responses given by sixty-six (36.5%) medical officers, notification forms were not available in their wards at the time of this study. The majority of the participants (101, 53.7%) mentioned they would still notify over the phone to the relevant MOH in the absence of notification forms while some participants (54, 28.7%) preferred to wait till notification forms are available.

**Table 2:** Doctors’ attitudes regarding leishmaniasis and notification system

	Strongly disagree	Disagree	Neutral	Agree	Strongly agree
**Attitudes about the disease**					
Leishmaniasis is an emerging disease in north central province	7(3.8%)	6(3.3%)	16(8.7%)	114(62.0%)	41(22.3%)
Early diagnosis and treatment is important in controlling Leishmaniasis.	10(5.5%)	09(4.9%)	01(0.5%)	99(54.1%)	64(35.0%)
Leishmaniasis can be eliminated from Sri Lanka.	10(5.5%)	17(9.3%)	35(19.1%)	95(51.9%)	26(14.2%)
**Attitudes about notification**					
Notification of Leishmaniasis is important.	12(6.6%)	1(0.5%)	4(2.2%)	76(41.5%)	90(49.2%)
All medical practitioners can notify diseases.	10(5.5%)	11(6.1%)	8(4.4%)	96(53.0%)	56(30.9%)
Current notification system is effective.	8(4.5%)	31(17.5%)	65(36.7%)	66(37.3%)	7(4.0%)
Barriers for timely notification are					
i) Unavailability of notification forms	19(11.7%)	52(31.9%)	32(19.6%)	53(32.5%)	7(4.3%)
ii) Heavy work load for medical officers	19(11.2%)	36(21.3%)	29(17.2%)	71(42.0%)	14(8.3%)
iii) Lack of staff to send the notification forms on time	19(12.1%)	55(35.0%)	28(17.8%)	45(28.7%)	10(6.4%)

**Table 3:** Doctors’ practices on leishmaniasis notification

	Yes	No	Not relevant
Have you suspected Leishmaniasis in any patient during last 8 years?	105(58.0%)	68(37.6%)	8(4.4%)
Have you notified any Leishmaniasis cases	35(19.4%)	134(74.4%)	11(6.1%)
Do you have notification forms at your ward/institute	98(54.1%)	66(36.5%)	17(9.4%)
Do you have notification forms at your private practice place/s	4(2.3%)	98(55.7%)	74(42.0%)
How do you notify in the absence of notification forms			
i) Do not notify	23(17.0%)	87(64.4%)	25(18.5%)
ii) Notify when notification forms are available	54(41.2%)	62(47.3%)	15(11.5%)
iii) Inform to the relevant MOH by a telephone call	101(67.3%)	37(24.7%)	12(8.0%)

Results of multiple linear regression analysis indicated that age and gender of the respondents were the only predictor variables significantly associated with the knowledge of leishmaniasis. (Unstandardized coefficient *B* = 0.106 (*P* = 0.03), *B* = 0.571 (*p* = 0.014) for age and gender respectively). Female doctors showed a better level of knowledge compared to males. Senior medical officers scored better than their junior colleges. But the correlation between these variables with the Knowledge score was weak. (Pearson correlation r, for age = 0.146, for gender 0.142 in both instance *P* < 0.05).

## Discussion

Leishmaniasis has now been identified as an emerging disease in Sri Lanka [14]. Emphasizing the need for controlling this disease Ministry of health, Sri Lanka declared it as a notifiable disease in 2008 [11]. As expected, doctors were more knowledgable about the clinical features, causative organism, disease transmission and the vector of Leishmaniasis compared to the studies done in the general public [15, 16]. But only 17% of respondents were aware that cutaneous leishmaniasis can be cured even without treatment. The practice of treating all the diagnosed patients of leishmaniasis may be the reason for this.

Although most of the participants had the idea that Leishmaniasis is an emerging disease in the country; just above half of the sample knew that the highest reported caseload of the country is from Anuradhapura district (the area in which they serve). This may be due to inadequate attention to publications of health ministry; weekly epidemiological reports and annual health bulletins that are available online, which gives updates regarding disease prevalence. It would be beneficial to establish a network among medical officers of the country through text messages or e-mails to keep them updated regarding infectious disease outbreaks. Such a system can even be useful in crisis situations as well.

In line with other studies regarding disease notification, there was no consensus among the responded doctors regarding the responsibility of filling the notification form and to whom a filled notification form should be handed over [13]. Only one-fifth of medical officers who suspected leishmaniasis had notified. This trend for leishmaniasis under-reporting was observed in previous studies conducted in Sri Lanka [12] and in other countries as well [17, 18]. Low rate of awareness on proper notification chain and the fact that disease notification is a legal requirement (as pointed out in the study findings) should be some of the factors responsible for this low notification rate. Several other studies have reported obstacles for disease notification similar to our findings such as infrastructure issues and lack of human resources [19–22], lack of time [19, 20, 23, 24] and lack of reporting forms/registers [24, 25]. These barriers were noted in the notification of other diseases like tuberculosis, malaria, and influenza as well [20, 26–28].

Notified data guides in distributing resources, monitoring and controlling the disease. Under notification and suboptimal attitudes regarding Leishmaniasis notification, can conceal the true disease burden and mislead the whole surveillance system. Notification switches on the contact tracing process and active case detection. Hence disease control is impossible to accomplish without proper notification.

The study confronted several limitations. Our sample was from one MOH area (and the majority of the participants are from one major tertiary care center) which can influence the external validity of the result. Although the room for acquiescence, habituation and sponsor bias was minimal, the possibility of over-reporting (social desirability bias) could not be excluded as there was no way of cross-checking what was reported is actually done. Despite all these limitations, we believe our findings provide a valuable source of information for policymakers. Estimation of under-reporting of other notifiable diseases is an important aspect that should be investigated in future researches.

## Conclusion

Even though more than 90% of medical officers participated in this study had good theoretical knowledge about leishmaniasis, notification of leishmaniasis is considerably inadequate though the study population is from an area with high disease burden. The findings of the study emphasize the need for regular awareness/training programs on notification chain and notifiable diseases. These programs can be incorporated into continuous medical education (CME) lecturers conducted in major hospitals. Establishing an electronic system (Eg: e-mail, online form system) for disease notification will enhance timely notification of all notifiable diseases while making a path to overcome some of the barriers of inadequate human resources and infrastructure. Responsible officials and stakeholders should take necessary actions to modify and monitor the notification system to control and eliminate leishmaniasis form Sri Lanka.

## Supplementary information

**Additional file 1.** A survey of knowledge, attitudes & practices related to Leishmaniasis among Medical officers in Nuwaragampalatha East MOH area, Anuradhapura. The questionnaire that was developed based on WHO fact sheet and Surveillance Case Definitions for Notifiable Diseases in Sri Lanka booklet is attached as a suplementary file.

## Data Availability

The datasets used and analysed during the current study are available from the corresponding author on reasonable request.

## Abbreviations

PCR: Polymerase chain reaction
SSG: Sodium stibogluconate
KAP: Knowledge, attitude, and practices
MOH: Medical officers of health
WHO: World Health Organization
MBBS: Bachelor of Medicine, Bachelor of Surgery
MD: Doctor of Medicine
SPSS: Statistical package for social sciences
CME: Continuous medical education
