# Supplementary material for: Is leishmaniasis adequately notified in Sri Lanka? A survey among doctors from an endemic district, Sri Lanka
Source: BMC Public Health. 2020 Jun 12;20:913. doi: 10.1186/s12889-020-09066-w (PMC7290071; doi:10.1186/s12889-020-09066-w)
Supplement: Supplementary file 1 — Additional file 1. A survey of knowledge, attitudes & practices related to Leishmaniasis among Medical officers in Nuwaragampalatha East MOH area, Anuradhapura. The questionnaire that was developed based on WHO fact sheet and Surveillance Case Definitions for Notifiable Diseases in Sri Lanka booklet is attached as a suplementary file. [file 12889_2020_9066_MOESM1_ESM.docx]

| S.Number: |  |  |  |  |
| --- | --- | --- | --- | --- |

**Survey of knowledge, attitudes & practices related to Leishmaniasis among Medical officers in Nuwaragampalatha East MOH area, Anuradhapura.**

Date: ……………………………..

| Teaching Hospital/Anuradhapura |  |
| --- | --- |
| Divisional hospital -Nelubewa |  |
| Divisional hospital –Nachchadoowa. |  |
| PMCU-Pubudupura |  |
| MOH- NPE |  |
| RDHS office Anuradhapura |  |
| PDHS office Anuradhapura |  |

Institution

**Part A- Socio-Demographic Data**

1, Age yrs

2, Sex

Male

Femaleee

3. Service experience: year/s

| Consultant |  |
| --- | --- |
| Grade Medical Officer |  |
| Intern Medical Officer |  |
| Registered Medical Officer |  |
| General Practitioner |  |

4. Current Position

5. Previous positions held:

**Part B-Assesment of Knowledge , Attitude and Practices.**

**1. Knowledge**

| **Q.No** | **Questions** | **Yes** | **No** | **Don’t know** |
| --- | --- | --- | --- | --- |
|  | **Knowledge about the disease.** |  |  |  |
| 1 | Leishmaniasis is caused by a parasite. |  |  |  |
| 2 | The causative agent of Leishmaniasis in Sri Lanka is *Leishmania donovani*. |  |  |  |
| 3 | The commonest manifestations of Leishmaniasis in Sri Lanka iscutaneous. |  |  |  |
| 4 | The vector of the Leishmaniasis is,  i) mosquito |  |  |  |
|  | ii) Sand fly |  |  |  |
|  | iii) tick |  |  |  |
| 5 | One of the commonest symptoms of cutaneous leishmaniasis is skin nodule or papule. |  |  |  |
| 6 | Skin lesions of cutaneous leishmaniasis is asymptomatic. |  |  |  |
| 7 | Highest disease burden of Leishmaniasis is reported from Anuradhapura District. |  |  |  |
| 8 | Cutaneous Leishmanis is cured without treatment |  |  |  |
| 9 | Diagnosis of cutaneous Leishmaniasis is mainly clinical |  |  |  |
| 10 | Treatments for Lesismanisis include  i) IV/IM Antibiotics |  |  |  |
|  | ii) localy applied steroids |  |  |  |
|  | iii) IV/IM Antimonials |  |  |  |
|  | **Knowledge about notification.** |  |  |  |
| 11 | Notification of leishmaniasis should be done  i)After laboratory confirmation. |  |  |  |
|  | ii)At clinical suspicion |  |  |  |
| 13 | Notification of leishmaniasis is a legal requirement. |  |  |  |
| 14 | Information regardingthe notification form will be entered in, |  |  |  |
|  | i)Notification register of the ward |  |  |  |
|  | ii)Notification register of the institute |  |  |  |
|  | iii)Notification register at the MOH office |  |  |  |
| 15 | From the hospital notification form will be sent to |  |  |  |
|  | i) Regional epidemiologist |  |  |  |
|  | ii) PHI |  |  |  |
|  | iii) Relevant MOH |  |  |  |
|  | iv) Patient’s home |  |  |  |

**2.Attitudes**

| **No** | **Questions** | **Strongly disagree** | **Disagree** | **Neutral** | **Agree** | **Strongly agree** |
| --- | --- | --- | --- | --- | --- | --- |
|  | **Attitudes about the disease.** |  |  |  |  |  |
| 16 | Leishmaniasis is an emerging disease in north central province |  |  |  |  |  |
| 17 | Early diagnosis and treatment is important in controlingLeishmaniasis. |  |  |  |  |  |
| 18 | Leishmaniasis can be eliminated from Sri Lanka. |  |  |  |  |  |
|  | **Attitudes about notification** |  |  |  |  |  |
| 19 | Notification of Leishmaniasis is important. |  |  |  |  |  |
| 20 | All medical practitioners can notify diseases. |  |  |  |  |  |
| 21 | Current notification system is effective. |  |  |  |  |  |
|  | Barriers for timely notification are |  |  |  |  |  |
| 22 | i) Unavailability of notification forms |  |  |  |  |  |
|  | ii) Heavy work load for medical officers |  |  |  |  |  |
|  | iii) Lack of the staff to send the notification forms on time |  |  |  |  |  |

**3. Practices**

| **No** | **Questions** | **Yes** | **No** | **Not relevant** |
| --- | --- | --- | --- | --- |
|  | **Practices about the disease.** |  |  |  |
| 23 | Have you suspected Leishmaniasis in any patient during last 8 years in the hospital? |  |  |  |
| 24 | When you suspect a patient has Leishmaniasis, you will; |  |  |  |
|  | i) Treat with antibiotics |  |  |  |
|  | ii)Do a dermatology referral |  |  |  |
|  | iii) Treat with locally applying steroids |  |  |  |
|  | iv) Advice your junior doctor to fill a notification form |  |  |  |
|  | v) Fill a notification form by your self |  |  |  |
| 25 | What you will do after filling the notification form |  |  |  |
|  | i) handover to your junior doctor |  |  |  |
|  | ii)handover to infection control nurse in the ward |  |  |  |
|  | iii) Send to relevant MOH after entering it to infection control register in the hospital. |  |  |  |
| 26 | Have you suspected Leishmaniasis in any patient during your private practice? |  |  |  |
|  | **Practices about the notifications.** |  |  |  |
| 27 | Have you notified any Leishmaniasis cases |  |  |  |
| 28 | Do you have notification forms at your word/institute |  |  |  |
| 29 | Do you have notification forms at your private practice place/s |  |  |  |
| 30 | How do you notify in the absence of notification forms  i) Do not notify |  |  |  |
|  | ii)Notify when notification forms are available |  |  |  |
|  | iii) Inform to the relevant MOH by a telephone call |  |  |  |

Your suggestions to improve notification of Leishmaniasis

………………………………………………………………………………………………………………………………………………………………………………………………………………………………………………………………………………………………………………………………………………………………………………………………………………………………………………………………………………………………………………………………………………………………………………………………………………………………
